# Bacterial nanocellulose production from naphthalene

**DOI:** 10.1111/1751-7915.13399

**Published:** 2019-05-14

**Authors:** Patricia Marín, Sophie Marie Martirani‐Von Abercron, Leire Urbina, Daniel Pacheco‐Sánchez, Mayra Alejandra Castañeda‐Cataña, Aloña Retegi, Arantxa Eceiza, Silvia Marqués

**Affiliations:** ^1^ Estación Experimental del Zaidín Department of Environmental Protection Consejo Superior de Investigaciones Científicas Calle Profesor Albareda, 1 Granada 18008 Spain; ^2^ Materials + Technologies Research Group (GMT) Department of Chemical and Environmental Engineering Faculty of Engineering of Gipuzkoa University of the Basque Country Pza Europa 1 Donostia‐San Sebastian 20018 Spain

## Abstract

Polycyclic aromatic compounds (PAHs) are toxic compounds that are released in the environment as a consequence of industrial activities. The restoration of PAH‐polluted sites considers the use of bacteria capable of degrading aromatic compounds to carbon dioxide and water. Here we characterize a new *Xanthobacteraceae* strain, *Starkeya* sp. strain N1B, previously isolated during enrichment under microaerophilic conditions, which is capable of using naphthalene crystals as the sole carbon source. The strain produced a structured biofilm when grown on naphthalene crystals, which had the shape of a half‐sphere organized over the crystal. Scanning electron microscopy (SEM) and GC‐MS analysis indicated that the biofilm was essentially made of cellulose, composed of several micron‐long nanofibrils of 60 nm diameter. A cellulosic biofilm was also formed when the cells grew with glucose as the carbon source. Fourier transformed infrared spectroscopy (FTIR) confirmed that the polymer was type I cellulose in both cases, although the crystallinity of the material greatly depended on the carbon source used for growth. Using genome mining and mutant analysis, we identified the genetic complements required for the transformation of naphthalene into cellulose, which seemed to have been successively acquired through horizontal gene transfer. The capacity to develop the biofilm around the crystal was found to be dispensable for growth when naphthalene was used as the carbon source, suggesting that the function of this structure is more intricate than initially thought. This is the first example of the use of toxic aromatic hydrocarbons as the carbon source for bacterial cellulose production. Application of this capacity would allow the remediation of a PAH into such a value‐added polymer with multiple biotechnological usages.

## Introduction

Polycyclic aromatic hydrocarbons (PAHs) are toxic compounds with a marked stability and resistance to degradation, which results in long‐term persistence in the environment, thus causing serious social concern. They are released into the environment as a result of industrial activity, although natural sources, such as hydrocarbon seeps, significantly contribute to the presence of these aromatic compounds in nature. Sixteen PAHs, among which naphthalene, are among the US EPA priority pollutants for their toxicity and potential mutagenic activity. Current remediation strategies for aromatic hydrocarbon‐polluted sites are based on the capacity of many bacteria to degrade natural and synthetic aromatic compounds to CO_2_ and water. On this basis, *in situ* remediation strategies rely on either providing nutrients to promote growth of the autochthonous microbial populations (biostimulation) or the addition of microbes with proven capacity to degrade a target contaminant (bioaugmentation). Bacteria able to degrade PAHs under aerobic conditions are ubiquitous and have been extensively characterized (Wackett and Hershberger, 2001). It is well established that the PAH biodegradation pathways involve aromatic mono‐ and dioxygenases for ring activation (hydroxylation) and ring cleavage as most relevant players (Vaillancourt *et al*., 2006; Ullrich and Hofrichter, 2007). Under aerobic conditions, the simplest PAH naphthalene undergoes a series of transformations to render salicylate, which is further oxidized to CO_2_ and water. The genes for the pathway are highly conserved and generally organized into two operons (Habe and Omori, 2003), as in the archetypal NAH7 plasmid: *nahABFCED* (from naphthalene to salicylate), and *nahGTHINLOMKJ* (from salicylate to pyruvate and acetyl‐CoA), in addition to a regulator, *nahR* (Yen and Gunsalus, 1982).

However, the availability of oxygen is often limited in hydrocarbon‐polluted sites. Stimulation of the autochthonous aerobic microbial communities by the presence of an external organic carbon source results in a high oxygen demand for aerobic degradation processes in initially aerobic sites, causing rapid oxygen depletion. This results in a decreasing oxygen concentration gradient and in the development of anoxic conditions (Lovley, 2003). Nitrate is generally the alternative electron acceptor at the edge between oxic and anoxic conditions (and also close to the surface of submerged sediments). In the oxic/anoxic interface at the fringes of polluted sites, the oxygen availability is likely to fluctuate (Kurt *et al*., 2016), and aerobic and nitrate‐reducing metabolisms generally coexist at the boundaries of polluted sites, where microaerophilic conditions prevail (Wilson and Bouwer, 1997; Yagi *et al*., 2010). Under microaerophilic conditions [defined as dissolved oxygen in the range of 0–2 mg l^−1^ (between 0% and 25% air saturation at 30°C)], minute amounts of oxygen suffice to promote the aerobic degradation of hydrocarbons (Yerushalmi *et al*., 2002; Táncsics *et al*., 2013). Interestingly, in strongly reduced polluted sediments where an anaerobic metabolism is expected, genes for aromatic monooxygenases (i.e. aerobic degradation pathways) have been shown to be highly abundant and no increase towards the oxidized plume leading edges has been observed, evidencing that the niche partitioning between aerobic and anaerobic degraders in the field is not fully resolved (Larentis *et al*., 2013).

Polycyclic aromatic hydrocarbon degradation has generally been analysed in the laboratory with pure cultures of microorganisms in the planktonic state. However, the situation in the environment is quite different because microbial processes usually involve biofilms composed of several coexisting bacterial groups that develop complex self‐organized supracellular structures, coordinated to create a suitable habitat for the community (Flemming *et al*., 2016). Bacterial growth forming biofilms is advantageous to the members of the community as compared to the planktonic state (Roder *et al*., 2016). The biofilm architecture is structurally supported by the extracellular polymeric substances (EPS) produced by the community members, which provide a number of advantages for the community, such as stability, cell–cell proximity favouring interactions and genetic exchange (Madsen *et al*., 2012; Ren *et al*., 2015), protection against stressors and predators, passive sorption of solutes, amongst other benefits. In fact, in hydrocarbon‐polluted sites, environmental bacterial isolates often only degrade a narrow range of PAHs, and cooperative processes involving a consortium of strains with complementary capacities are frequently observed (Bouchez *et al*., 1995; Wang *et al*., 2016).

Biofilms also play a relevant role in hydrocarbon biodegradation by making these hydrophobic compounds more accessible to microorganisms (Johnsen *et al*., 2005; Heipieper *et al*., 2007; Espinosa‐Urgel and Marqués, 2017). Biofilm formation at the hydrocarbon–water interface is believed to increase the flux of hydrocarbons to the cells by diminishing the distance between the carbon source and the cell surface (Wick *et al*., 2001; Wackett, 2015). Moreover, the EPSs in biofilms constitute an extracellular matrix that influences hydrocarbon utilization (Ennouri *et al*., 2016). This extracellular matrix generally includes proteins, nucleic acids and exopolysaccharides, and its role in different bacteria and under different environmental conditions has been addressed (Hinsa *et al*., 2003; Chang *et al*., 2007; Martínez‐Gil *et al*., 2013). Different hypotheses have been proposed to explain the advantages of biofilm EPSs in hydrocarbon degradation: adsorption of the hydrophobic substrates to the matrix, retention of enzymes, location of intermediate products and biosurfactants close to the cell, and an increase in the cell adhesion properties have been suggested as possible mechanisms enhancing the diffusion of hydrocarbons from the organic phase to the cell (Harms *et al*., 2010). Accordingly, a number of hydrocarbonoclastic bacteria have been shown to develop biofilms when grown on hydrocarbon compounds as the sole carbon source (Golyshin *et al*., 2002; Mounier *et al*., 2014). In many cases, the biofilm was only produced in the presence of hydrophobic degradable substrates, whereas the loss of the biofilm forming capacity implied limited hydrocarbon degradation (Johnsen and Karlson, 2004; Klein *et al*., 2008). Hydrocarbon degradation dependent on biofilm formation has been observed both in real marine hydrocarbon spill incidents, where biodegradation was detected in bacterial flocs associated with oil droplets, and in laboratory experiments (Hazen *et al*., 2010; Netzer *et al*., 2018). Nevertheless, the nature of the compounds in the extracellular matrix of the biofilms developed in the presence of hydrocarbons and other hydrophobic compounds is poorly understood, and their function is largely unknown. Furthermore, little is known about the mechanism through which biofilm development stimulates hydrophobic substrate assimilation.

Amongst the bacterial secreted EPSs, cellulose (bacterial cellulose, BC) emerges as a nanomaterial that has attracted substantial interest due to its unique physicochemical and mechanical properties, and it can be used for a variety of commercial applications including textiles, cosmetics, medical and food products (Gama *et al*., 2013; Abitbol *et al*., 2016). The BC producers *par excellence* are acetic acid bacteria, and among them *Gluconacetobacter xylinum* (renamed as *Komagataeibacter xylinum*) and *Gluconacetobacter hansenii* are among the most efficient and better‐characterized producers (Florea *et al*., 2016). Recently, a number of interesting cellulose‐producing strains belonging to different taxa, including several members of the *Enterobacterales*, have been identified as promising cellulose producers (Jedrzejczak‐Krzepkowska *et al*., 2016). In a previous study, we enriched a naphthalene degrading, biofilm forming, microbial community from samples collected at the oil–water interface of a hydrocarbon‐polluted aquifer, using microaerophilic conditions resembling those naturally found in real polluted sites in the environment. Oxygen limitation was the main factor driving the community structure, selecting for strains that were undetectable in the initial material (Martirani‐Von Abercron *et al*., 2017). Interestingly, this enrichment evidenced the function of the biofilm to favour gene transfer, leading to the blooming of a naphthalene degrading strain able to grow at very low oxygen levels and with a striking capacity to develop a thick biofilm, probably intended to locate the cells at the appropriate distance from the toxic carbon source. In this study, we have analysed the nature and properties of the biofilm and the capacity of the strain, *Starkeya* sp. strain N1B, to produce this biofilm at a distance from the naphthalene crystal used as the sole carbon source. Preliminary results suggested the EPS could be mainly composed of cellulose, thus opening the possibility of future exploitation of the strain in the recycling of hydrocarbon wastes into a biotechnologically valuable nanomaterial.

## Results and discussion

### *Starkeya* sp. strain N1B produces a structured biofilm when growing on naphthalene

**Figure 1:**
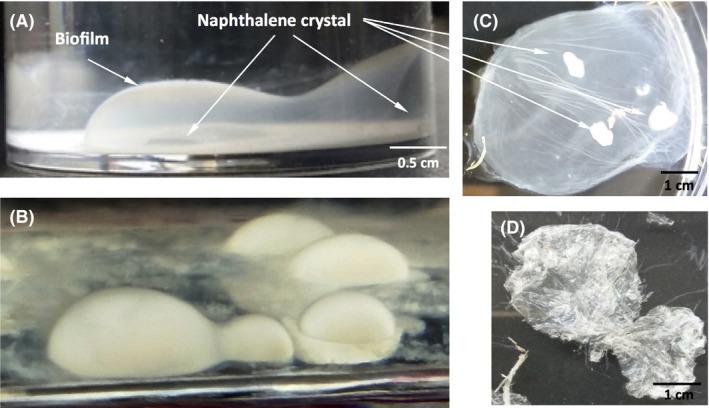
Biofilm developed by strain *Starkeya* sp. strain N1B when growing with naphthalene crystals as the sole carbon source. Cultures in a flask containing two (A) or five naphthalene crystals (B). Appearance of a wet cellulose pellicle after collection, with visible naphthalene remnants (C) and after lyophilization (D).

We previously isolated *Starkeya* sp. strain N1B for its ability to grow with naphthalene crystals as sole carbon source (Martirani‐Von Abercron *et al*., 2017). We sequenced the genome of N1B strain, which resulted in 57 contigs that covered the expected 4.79 Mb of the genome. Although the strain was first assigned to the species *S. novella*, a comparison of its draft genome with that of the type strain *Starkeya novella* using JSpecies (Richter *et al*., 2016) gave an ANIb value of 92.7, below the cut‐off value of 95.5, which placed our strain at the limit of genome homology for species assignation. We thus decided to only assign the strain at the genus level. When grown with naphthalene in static bottles under microaerophilic conditions, the strain developed a thin biofilm initially located at 0.5–1 cm around the naphthalene crystal (Fig. 1A), so that an opaque structure with the shape of a half‐sphere was observed in the culture medium over the naphthalene crystal. A progressive increase in biofilm thickness occurred with time, leading to the biofilm finally invading the empty space between the initial biofilm pellicle and the naphthalene crystal, which was progressively consumed (Fig. 1B). Although it took between four to six weeks for a consistent biofilm to develop, the incipient biofilm was already eye‐visible after one week's growth. When the strain was cultured under full aerobic conditions in Erlenmeyer flasks shaken at 100 rpm, the strain grew forming aggregates attached to the naphthalene crystal. Finally, when the naphthalene was provided dissolved in heptamethylnonane (HMN) in a layer covering the surface of the static medium, the biofilm developed as a flat sheet below the hydrocarbon layer that progressively sank as it developed as a thick biofilm (Fig. S1A).

When the biofilm was collected from the culture medium with the naphthalene crystals, it appeared as a circular gelatinous pellicle, sometimes including remnants of the naphthalene crystals used for growth (Fig. 1C). As expected, the size and thickness of the pellicle was reduced after lyophilization (Fig. 1D).

### The biofilm structure is made up of nanocellulose fibres

**Figure 2:**
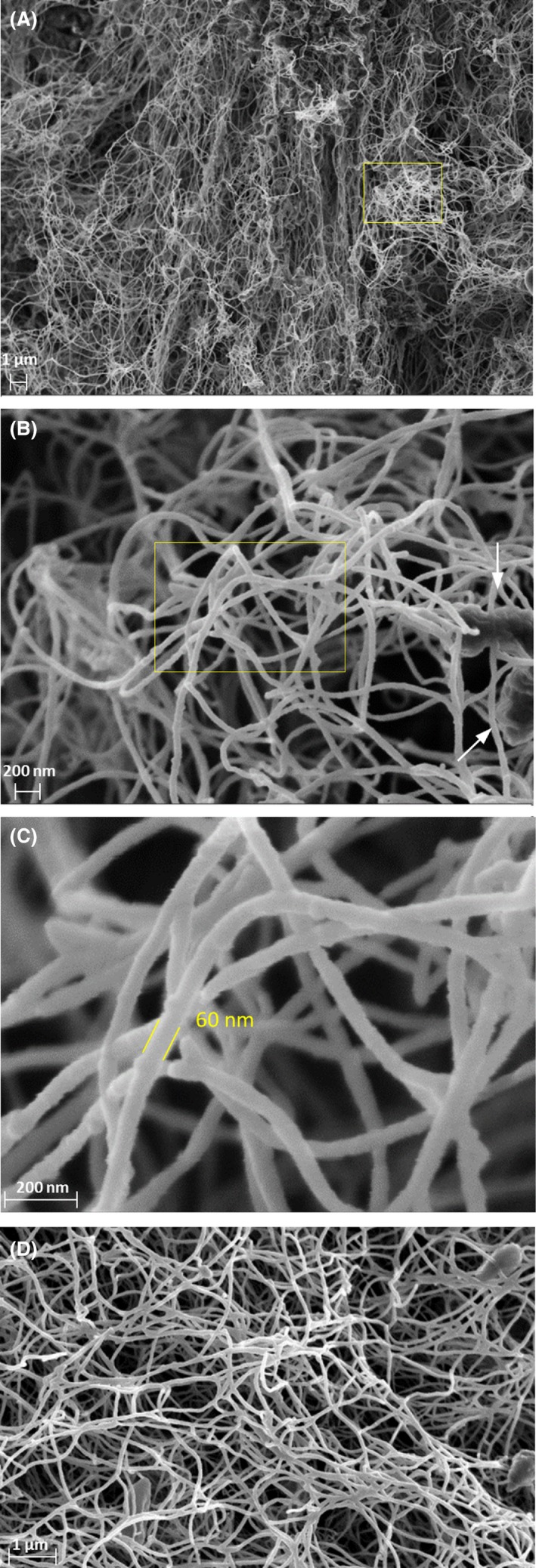
SEM images of *Starkeya* sp. strain N1B biofilm formed during growth on naphthalene (A, B and C) and glucose (D). The magnified region in each image is indicated by a yellow square. The estimated diameter size of the nanofibers is shown in C. White arrows in B indicate the presence of bacterial cells.

As a first approach to characterizing the structure of the biofilm, we used scanning electron microscopy (SEM) to visualize biofilm samples prepared from cultures grown on naphthalene crystals. Figure 2A–C shows the presence of a tridimensional network made of fibrils of an average cross‐sectional dimension of 60 nm and several μm length, which is characteristic of bacterial nanocellulose (Klemm *et al*., 2005; TAPPI, 2011). The fibre network left large pores between the fibrils. The number of visible bacterial rods of approximately 1 μm long was low, and the rods were submerged in the fibril network, touching one or several fibrils. The cellulosic nature of the fibril was further substantiated by a GC‐MS analysis of acid‐hydrolysed biofilm material, which was exclusively composed of glucose.

**Figure 3:**
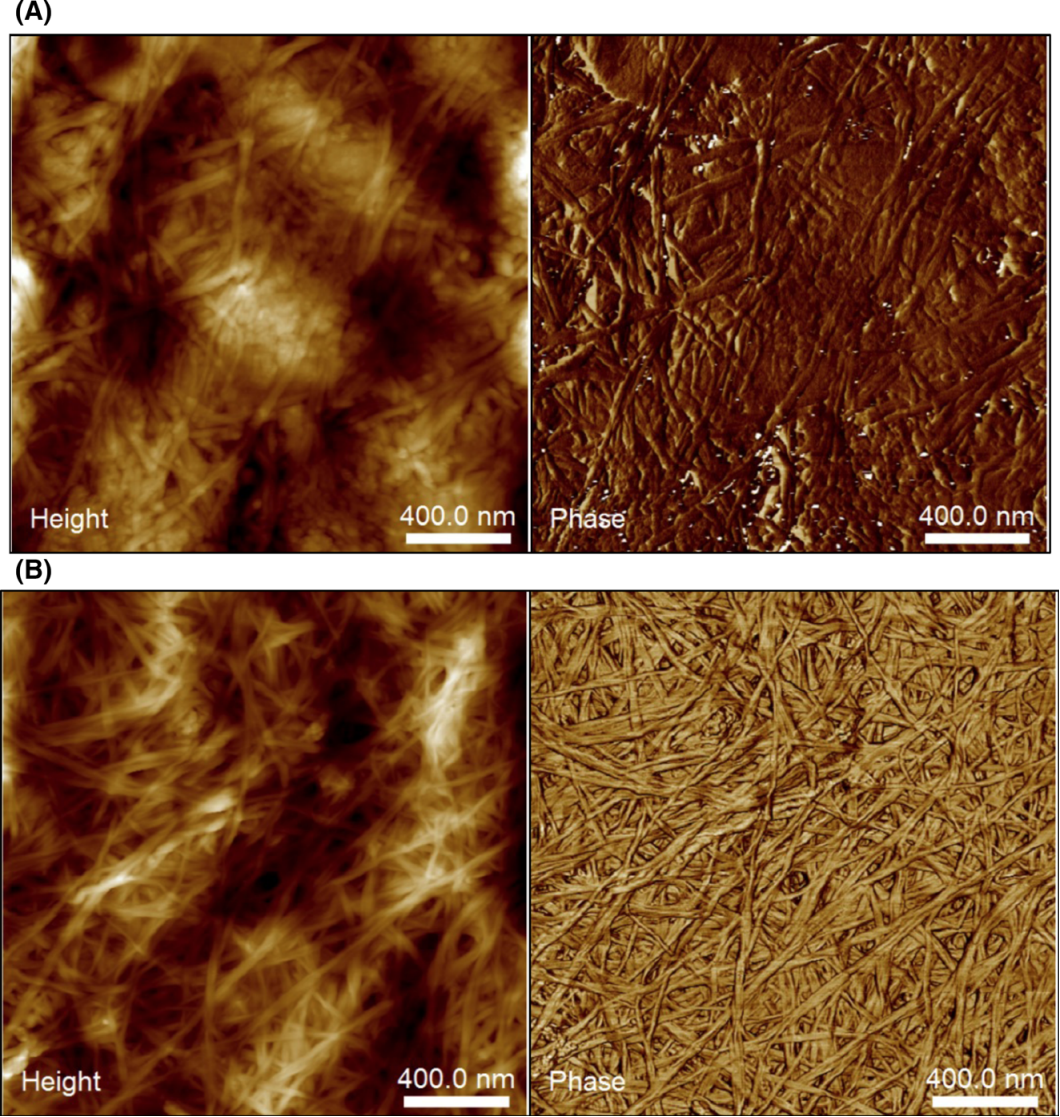
AFM height (left) and phase (right) images of the *Starkeya* sp. strain N1B biofilm formed during growth on naphthalene (upper panels) and glucose (lower panels). Fibrils were purified with 0.5% aqueous KOH solution, thoroughly washed with water to neutrality and lyophilized.

We used atomic force microscopy (AFM) to characterize the morphology of the cellulose biofilm surface. Figure 3 shows the AFM height of the lyophilized cellulose material obtained from cultures grown on naphthalene crystals and treated with 0.5% KOH to eliminate bacterial cells. We observed a random orientation of the nanofibrils in the network, which intertwined with each other to form different compact layers, as already observed in the SEM micrographs, apparently with some amorphous regions (Fig. 2). The diameter of the nanofibrils estimated from AFM height (average of 20 measurements) was 40 ± 8 nm. AFM also confirmed that the fibril length was of several μm.

Strain N1B was also capable of cellulose synthesis when directly grown on glucose as the sole carbon source. In this case, a consistent and flat biofilm made of superimposed layers developed close to the bottom of the culture bottle when growing statically (Fig. S1B). A SEM image of the biofilm showed a similar structure of that observe with naphthalene (Fig. 2D). AFM of the material also showed the tridimensional structure typical of bacterial cellulose, where nanofibrils were randomly oriented, although in this case they showed a more homogeneous and reticulated conformation than the material formed from naphthalene. The nanofibrils had on average a 33 ± 8 nm diameter.

**Figure 4:**
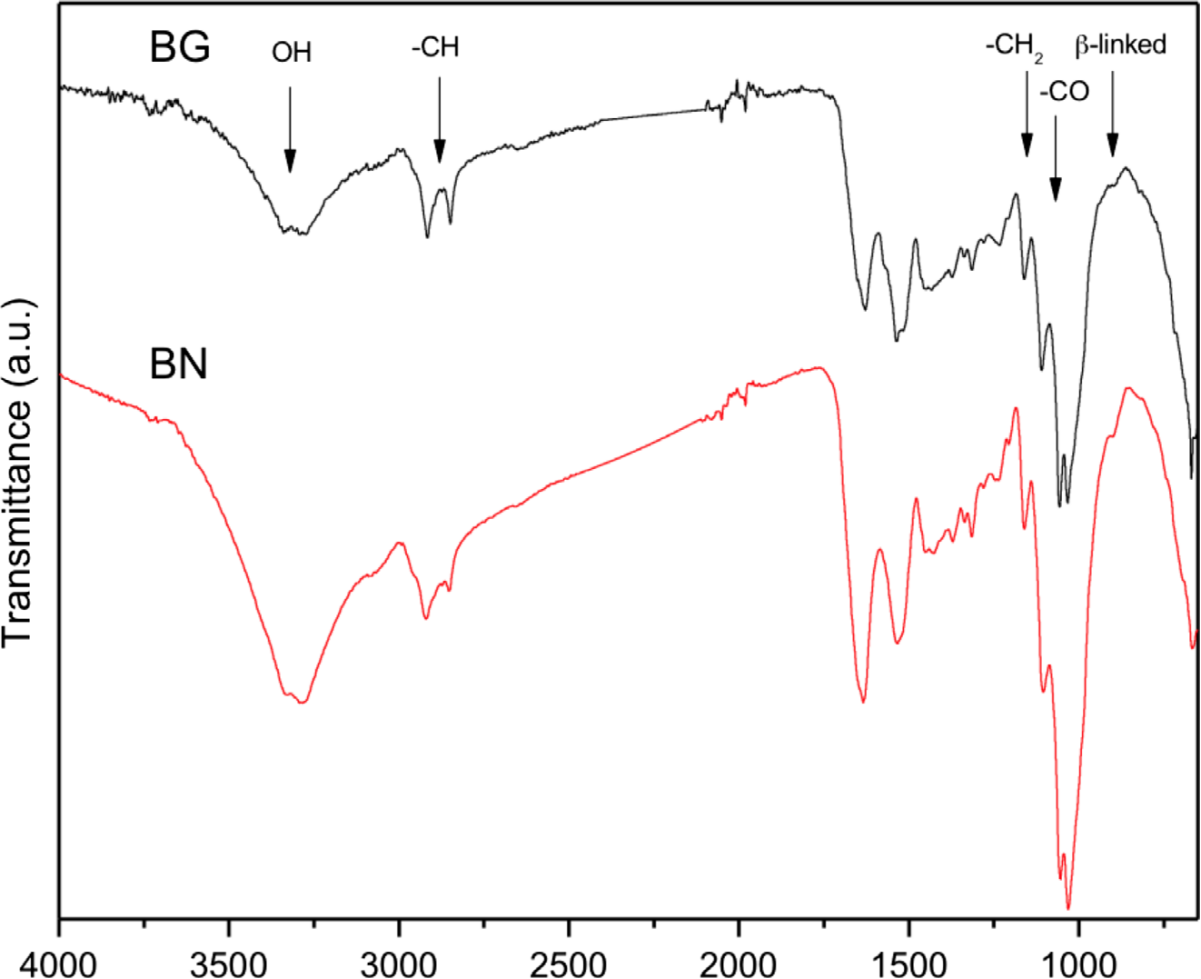
ATR‐FTIR spectra of cellulose membranes produced by *Starkeya* sp. strain N1B growing on naphthalene crystals BN and on glucose (BG).

Attenuated total reflectance–Fourier transform infrared spectroscopy (ATR‐FTIR spectroscopy) showed that the material obtained from the cultures on both naphthalene and cellulose had the characteristic profile of bacterial cellulose (Fig. 4) (Nelson and O'Connor, 1964; Yamamoto *et al*., 1996; Kondo *et al*., 2016; Urbina *et al*., 2017). The typical bands of bacterial cellulose were observed: a broad band near 3200 cm^−1^ corresponding to OH stretching vibrations; bands at 2900 and 2880 cm^−1^ corresponding to CH stretching vibrations; the bands at 1460 and 1250 cm^−1^ corresponding to CH_2_ bending vibrations; bands at 1170 and 1050 cm^−1^, corresponding to the vibration of C‐O‐C bonds of glycosidic bridges; and a band at 897 cm^−1^ characteristic of β‐linked glucose‐based polymers was also observed. These spectra are compatible with type I cellulose, although the slight shift towards lower wave numbers could reflect the presence of some type II cellulose (Yue *et al*., 2013). In addition, two strong bands at 1530 and 1630 cm^−1^, which are not characteristic of cellulose, were clearly visible in both samples. Bands at 1650 cm^−1^ are generally ascribed to water ‐OH groups contained within the structure, which would hardly be the case here because the samples were lyophilized and dried at 60°C for 24 h. This region is also related to compounds with aromatic groups and unsaturated C‐C bonds. However, only the cultures with naphthalene as carbon source contained this aromatic compound, which ruled out the presence of aromatics as cause of the abnormality of the spectra. Since both culture media contained MOPS [3‐(*N*‐morpholino)propanesulfonic acid] as the biological buffer, which is known to strongly interact with water molecules and alter the hydration layer surrounding polymers such as proteins (Taha *et al*., 2011; Gupta *et al*., 2015), the presence of 30 mM MOPS in the cultures might explain the unexpected bands at 1530 and 1630 cm^−1^. The sulfone's oxygen atom of MOPS molecule could probably form strong hydrogen bonds with the hydroxyl groups of the cellulose surface, modifying the resulting spectrum. Alternatively, these bands may reflect the contamination of the KOH‐treated samples with bacterial cell remains (proteins, nucleic acids and lipids), which generally appear as broad bands in the wave‐number range 1750–1400 cm^−1^ (Fuller *et al*., 2018). The presence of either MOPS or some other compound in the medium during cellulose synthesis seems to have altered the packaging and properties of the resulting material. This is especially true for the cellulose produced from naphthalene, whereas the cellulose membranes obtained from glucose cultures presented a canonical type I cellulose X‐ray diffractogram (not shown), the peaks were poorly defined in the samples originated from naphthalene and showed a very low crystallinity index, closer to amorphous cellulose, as already suggested in the AFM images.

### A composite genetic complement for naphthalene degradation in strain N1B

**Figure 5:**
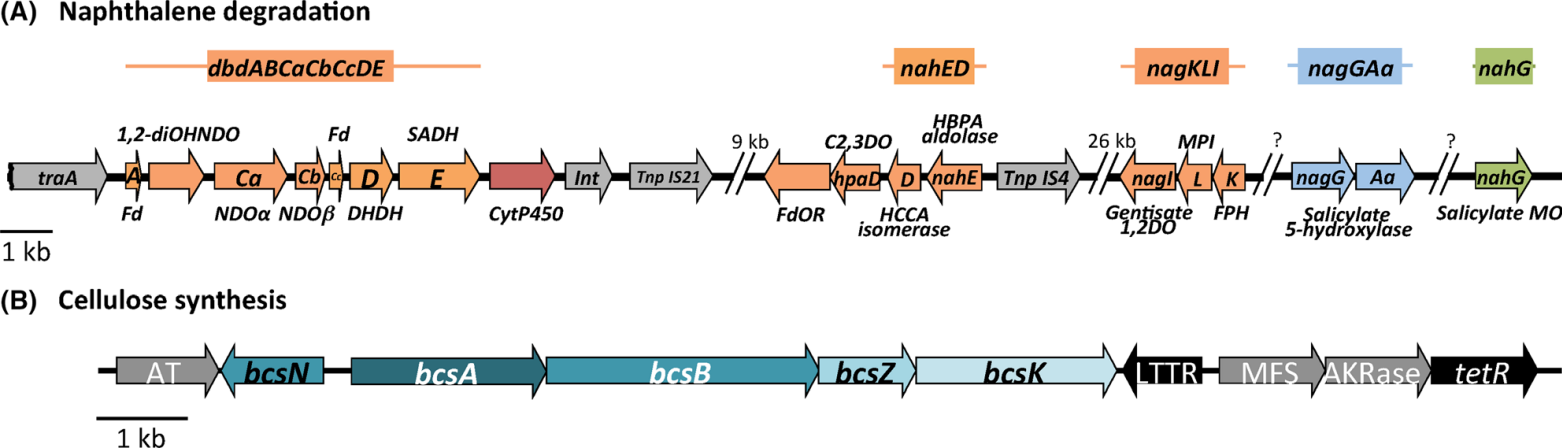
Relevant gene clusters in *Starkeya* sp. strain N1B genome. The genes are presented as arrows with the gene name inside. A. Gene clusters for the degradation of naphthalene. Genes with the same colour were present in the same contig; the kb numbers between clusters show the distance between the clusters in the contig sequence. The gene cluster name ascribed in the deposited sequences is shown above each cluster. Transfer elements are coloured in grey. The protein encoded by each gene is indicated above or below the corresponding gene. The abbreviations are 1,2‐diOHNDO, 1,2‐dihydroxynaphthalene dioxygenase; C1,2DO, catechol 1,2‐dioxygenase; DHDH, dihydrodiol dehydrogenase; DO, dioxygenase; Fd, ferredoxin; FdOR, ferredoxin oxidoreductase; FPH, fumarylpyruvate hydrolase; HBPA,* cis‐o*‐hydroxybenzalpyruvate; HCCA, 2‐hydroxy‐2*H*‐chromene‐2‐carboxylic acid; Int, integrase; MO, monooxygenase; MPI, maleylpyruvate isomerase; NDO, naphthalene dioxygenase; SADH, salicylate dehydrogenase; Tnp, transposon;. B. Gene cluster for cellulose biosynthesis. The genes known to be involved in cellulose synthesis are shown in blue–green colours; the neighbour genes unrelated to cellulose synthesis are shown in grey; the genes with homology to transcriptional regulator genes are in black. AT, acyl transferase; AKR, aldo/keto reductase family protein; MFS, major facilitator family protein; LTTR, LysR‐type transcriptional regulator.

In a previous study, we detected the presence in N1B strain of three genes with homology with genes of *Xanthobacter polyaromaticivorans dbd* operon for dibenzothiophene and naphthalene degradation (Hirano *et al*., 2006; Martirani‐Von Abercron *et al*., 2017). To identify the complete genetic complement for naphthalene degradation, we used local blast to locate the previously identified *dbd* cluster in the 166 kb contig‐20, flanked by insertion elements and several transfer genes (Fig. 5A). The enzymes encoded in the cluster would account for the transformation of naphthalene into 2‐hydroxy‐2*H*‐chromene‐2‐carboxylic acid (HCCA) and also for the conversion of 2‐hydroxybenzaldehyde into salicylate. A gene coding for a putative Cyt450 of unknown function was also present in the cluster (Fig. S2, Table 1). The genes for the transformation of HCCA into 2‐hydroxybenzaldehyde were absent, as were the genes for the further degradation of salicylate to TCA cycle intermediates. We were able to map a mini‐Tn5 random mutant of strain N1B selected for its failure to grow with naphthalene as sole carbon source in a gene with homology to *nahD*, coding for a HCCA isomerase. The gene was also located in contig‐20 but at 9 kb from the *dbd* cluster (Fig. 5A). It was flanked on one side by a gene with homology to *nahE* gene, coding for a *cis‐o*‐hydroxybenzalpyruvate aldolase, followed by an insertion sequence. These two genes thus completed the pathway from naphthalene to salicylate (Fig. S2). In naphthalene degrading bacteria, the further degradation of salicylate can proceed either through catechol or through gentisate as intermediates (Habe and Omori, 2003). At the other side of *nahD*, we found a gene with homology to catechol 2,3‐dioxygenase, followed by a ferredoxin oxidoreductase gene. Furthermore, a local blastp search in the annotated strain N1B contigs identified, in contig‐14, a protein with 31% identity with a salicylate monooxygenase from *Pseudomonas* (*nahG* gene), suggesting that catechol might be metabolized to 2‐hydroxymuconic semialdehyde and thus naphthalene degradation may proceed via catechol. However, growth of the strain on catechol was unclear. Because the strain normally grows forming insoluble aggregates, we used a cellulose deficient mutant (see below) to measure growth on catechol. The results clearly showed that the strain was incapable of growth with catechol as the carbon source.

**Table 1:** Products of the genes involved in naphthalene degradation in strain N1B

Gene name	Proposed gene function	Contig	Protein product closest relative^a^	% ID
*nahG*	Salicylate 1‐monooxygenase	14	FAD‐dependent oxidoreductase, *Ancylobacter rudongensis*	78
*nagG*	Salicylate 5‐hydroxylase alpha subunit	15	Aromatic ring‐hydroxylating dioxygenase subunit alpha, *Bosea* sp.	92
*nagAa*	Fd‐NADP oxidoreductase	15	Hybrid‐cluster NAD(P)‐dependent oxidoreductase, *Ancylobacter* sp.	84
*nahD*	2‐hydroxychromene‐2‐carboxylate isomerase	20	2‐hydroxychromene‐2‐carboxylate isomerase, *Puniceibacterium* sp.	64
*hpaD*	Catechol 2,3 dioxygenase	20	HpaD family, catechol 2,3 dioxygenase, *Rhodobacteraceae*	81
*nahE*	*cis‐o*‐hydroxybenzalpyruvate (HBPA) aldolase	20	Aldolase Class I, *Rhodobacteraceae*	79
*nagI*	Gentisate 1,2 dioxygenase	20	Gentisate 1,2‐dioxygenase, *Ensifer sojae* plasmid	94
*nagL*	Maleylpyruvate isomerase	20	Maleylacetoacetate isomerase, *Ensifer sojae*	84
*nagK*	Fumarylpyruvate hydrolase	20	Fumarylacetoacetate (FAA) hydrolase family protein, *Ensifer sojae*	84
*dbdA*	2Fe‐2S ferredoxin	20	DbdA, *Xanthobacter polyaromaticivorans*	100
*dbdB*	Gentisate 1,2‐dioxygenase	20	DbdB, *Xanthobacter polyaromaticivorans*	100
*dbdCa*	Benzene 1,2‐dioxygenase subunit alpha	20	DbdCa, *Xanthobacter polyaromaticivorans*	99
*dbdCb*	Benzene 1,2‐dioxygenase subunit beta	20	DbdCb, *Xanthobacter polyaromaticivorans*	98
*dbdCc*	Naphthalene 1,2‐dioxygenase, ferredoxin component	20	DbdCc, *Xanthobacter polyaromaticivorans*	99
*dbdD*	Cis‐naphthalene dihydrodiol dehydrogenase	20	DbdD, *Xanthobacter polyaromaticivorans*	98
*dbdE*	Salicylaldehyde dehydrogenase	20	DbdE, *Xanthobacter polyaromaticivorans*	98
cytP450	Cytochrome P450	20	Cytochrome P450, *Rhodobacteraceae*	32

**a**. When the function in nr database was unclear, the blastp search was done in the UniProtKB/Swiss‐Prot database.

On the other hand, about 12 kb upstream from the *nahED* cluster we identified a gene cluster coding for three enzymes involved in the degradation of gentisate: gentisate 1,2‐dioxygenase (*nagI*), maleylpyruvate isomerase (MPI, *nagL*) and fumarylpyruvate hydrolase (FPH, *nagK*) (Fig. 5A, S2). Several insertion sequences were also present in the vicinity of this cluster. A search for putative oxygenases in the automatically annotated draft genome of strain N1B identified in contig‐15 a gene cluster coding for homologues of salicylate 5‐hydroxylase subunit NagG and the accompanying ferredoxin oxidoreductase subunit NagAa. Interestingly, this latter protein showed the same two domains of NagAa from *Rasltonia* sp. strain U2, a ferredoxin reductase (FNR) Rieske‐type iron–sulphur binding domain and a fer2 2Fe‐2S iron–sulphur cluster binding domain, although in a reverse organization, i.e. the fer2 domain in the N‐terminal end and the FNR iron–sulphur domain in the C‐terminal end of the protein. We could not find in the vicinity any gene coding for either a ferredoxin (*nagAb*) or for NagH, a protein of unknown function generally conserved in other gentisate 1,2‐dioxygenase clusters (Fuenmayor *et al*., 1998). Assuming that this latter protein is dispensable and that other ferredoxins present in the genome could replace NagAa, this cluster would allow the transformation of salicylate into gentisate, thus closing the alternative pathway from salicylate to TCA cycle intermediates. In agreement with these findings, both strain N1B and its *bcsA* mutant were able to consistently grow with gentisate as the only carbon source. We searched for the presence of the naphthalene degradation genes described above in the only *Starkeya* genome available to date in the databases (Kappler *et al*., 2012). Only the genes *nahG*,* nagG* and *nagAa* had homologues with more than 90% identity in the *Starkeya novella* genome. This, together with the different origin of the degradation clusters annotated in this study (Table 1) and the abundance of insertion and transfer sequences in their neighbourhood, suggest that during the enrichment process under microaerophilic conditions with naphthalene as the sole carbon source, the strain had progressively acquired the functions required for complete naphthalene degradation through horizontal gene transfer.

Finally, it is worth noting that the genetic complement required for the transformation of TCA cycle intermediates into UDP‐glucose, the substrate for cellulose synthase, was present in the draft genome of strain N1B.

### Genetic determinants for cellulose synthesis in strain N1B

In contig‐16 of strain N1B genome, we identified a gene cluster coding for five proteins with homology with enzymes involved in cellulose synthesis (Fig. 5B, Table 2). This cluster was also present in the only *Starkeya* genome available in the databases, with more than 95% identity at the protein level. The gene content and organization resembled that of type IIIb *bcs* operons characteristic of some *Alphaproteobacteria* such as *Methylobacterium extorquens* (Römling and Galperin, 2015). The cluster included the genes for the two main catalytic subunits of cellulose synthase, BcsA and BcsB. BcsA contained the expected eight transmembrane segments and a PilZ domain for c‐di‐GMP binding (Morgan *et al*., 2014), whereas BcsB had a signal peptide in the N‐terminal end, and a single transmembrane segment at the C‐terminal end of the protein, which is believed to anchor this periplasmic protein to the membrane (Morgan *et al*., 2013). The BcsC subunit, a tetratricopeptide repeat (TPR)‐containing scaffold protein which is believed to interact with the peptidoglycan layer through its N‐terminal end and export the polymer through a channel in the outer membrane formed by the β‐barrel porin at its C‐terminal end (Whitney and Howell, 2013), was not present in the cluster. Instead, the cluster included a homologue to *bcsK* gene, encoding a different TPR‐containing protein that is thought to have the same function as BcsC (Römling and Galperin, 2015). In addition to these three core genes, the strain N1B *bcs* operon included a gene coding for a homologue of a periplasmic endoglucanase of family 8 glycoside hydrolases (GH8) annotated as BcsZ (Mazur and Zimmer, 2011). The gene included the expected signal peptide at its N‐terminal end, confirming its suggested periplasmic location (Ahmad *et al*., 2016). The function of BcsZ in cellulose biosynthesis is unclear, and its conserved presence in different *bcs* operons seems contradictory, considering its suggested endoglucanase activity. However, it has recently been shown to be required for c‐di‐GMP‐dependent regulation of cellulose biosynthesis (Castiblanco and Sundin, 2018). Finally, a gene with homology to *bcsN*, only present in type III *bcs* operons, was found upstream and divergent to *bcsA*. The function of this subunit, predicted to be located in the periplasm, is unknown.

**Table 2:** Products of the genes in the cellulose synthesis cluster in strain N1B

Protein	Closest relative^a^	Accession	%ID
BcsN	BcsN [*Bosea* sp. 117]	WP_051661218	56
BcsA	BcsA [*Bosea* sp. 117]	WP_029354417.1	73
BcsB	BcsB [*Bosea* sp. 117]	WP_051661219.1	54
BcsZ	Endoglucanase [*Bosea* sp. 117]	WP_029354420.1	62
BcsK	Tetratricopeptide repeat protein [*Microvirga* sp. CCBAU 65841]	WP_114943116.1	45

a The closest relative in the databases was in all cases a gene product from *Starkeya novella*, with more than 95% identity, which has not been considered here.

Flanking this cluster, we found on one side a gene coding for an acyl transferase with 73% identity with a homologous gene in *Ancylobacter rudongensis*. Interestingly, a comparison of the gene neighbourhood of the acyl transferase gene in *A. rudongensis* and strain N1B evidenced that the two flanking sequences of the *bcsA* cluster were conserved and contiguous in *A. rudongensis* (Fig. S3), suggesting an early lateral gene transfer event of the sequence spanning form *bcsN* to the aldo/keto reductase gene. The role of the last three genes in this sequence, coding for a LysR‐type transcriptional regulator (LTTR), a major facilitator superfamily protein and the aldo/keto reductase, apparently unrelated to cellulose biosynthesis, needs to be clarified. No homologue of these genes in the databases has been described to be involved in cellulose synthesis. Finally, it is worth mentioning that the cellulose synthesis cluster of *Agrobacterium fabrum* includes BcsL, a protein with acyl transferase activity. Although the acyl transferase encoded by the gene preceding the *bcs* cluster of strain N1B has no evident homology with BcsL, the possible role of this gene in cellulose synthesis needs to be elucidated.

### Biofilm formation is not essential for growth on naphthalene

**Figure 6:**
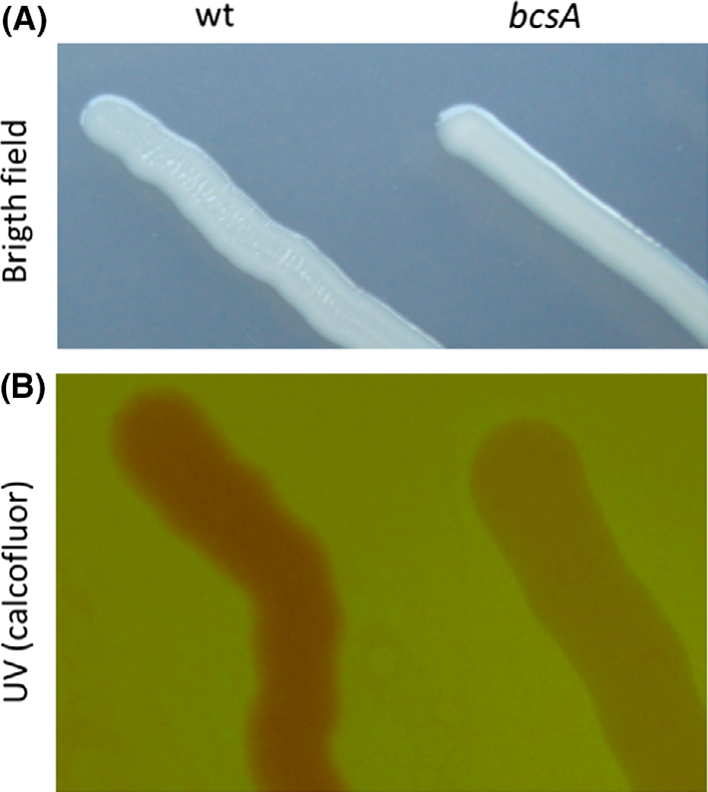
Calcofluor staining of wild type and *bcsA* mutant of strain N1B. (A) Bright‐field view of wild‐type (left) and *bcsA* mutant (right) strains growing on WMM plus glucose. Whereas the wild‐type strain shows a crinkly surface, the *bcsA* mutant surface is flat. (B) Wild‐type N1B strain fluoresced on calcofluor‐glucose agar plates (left) in comparison with the *bcsA* mutant strain (right). (Inverted image colours are presented).

Because of the precise architecture of the biofilm around the naphthalene crystal that places the bacterial cells at a distance from the potentially toxic carbon source, we considered the possibility that biofilm formation and cellulose synthesis are essential for growth on naphthalene. To test this hypothesis, we generated a deletion mutant of *bcsA* in strain N1B genome using reverse genetics. The resulting mutant strain, N1BΔbcsA, was able to grow on rich medium although it did not form the typical aggregates observed in the wild‐type strain. The incapacity of N1BΔbcsA to produce cellulose was evidenced by the morphology of the strain when grown on agar plates with glucose as the carbon source, whereas the N1B wild‐type strain showed some crinkles on the colony surface, the mutant strain was completely flat (Fig. 6A). This was confirmed by the absence of calcofluor (CF) staining of the mutant strain as compared to the wild type (Fig. 6B). The strain was also capable of growth with naphthalene crystal as the sole carbon source, although it grew as planktonic cells and did not form the biofilm around the crystal observed in the wild type. Because the wild‐type strain formed sturdy aggregates when grown on any carbon source, it was impossible in our hands to reliably quantify its growth. To compare the growth rate of the wild‐type and *bcsA* mutant strains with naphthalene as the sole carbon source, we determined naphthalene consumption in the two strains using naphthalene dissolved in HMN placed in an overlaying layer above the culture medium. Controls of non‐inoculated medium were used to quantify naphthalene evaporation in these conditions. The results showed that naphthalene consumption by the wild‐type and mutant strains was similar, and no significant difference was observed. This result indicates that at least when provided from a non‐aqueous liquid solution, the capacity to produce a cellulose biofilm does not entail any advantage to the strain to use naphthalene as the carbon source. In biodegradation experiments, HMN is used to keep naphthalene concentrations in the aqueous phase below saturation (approximately 35 μM in this case) and minimize the possible toxicity of the PAH (Ghoshal *et al*., 1996), whereas naphthalene concentration reaches saturating concentrations (approximately 200 μM) when provided from crystals. It is a fact that the *Starkey* sp. strain N1B does produce a complex biofilm structure when grown with naphthalene crystals as sole the carbon source, which suggests that it must provide some kind of benefit to the cell, especially considering the high cost of cellulose production. At saturating naphthalene concentrations in the aqueous phase, the proper location of the cells close to but at a distance from the crystal might be crucial for optimizing naphthalene uptake by the cells whilst protecting them from a possible toxic effect, which would not be required when the final naphthalene concentration was far below saturation as is the case with naphthalene dissolved in HMN.

## Concluding remarks

As reported in the literature, the production of bacterial cellulose generally demands culture media rich in carbon sources to be efficiently converted into the polymer structure, and a high diversity of waste products have been assayed as substrates for cellulose production (Jozala *et al*., 2016). However, the use of toxic aromatic hydrocarbons as the carbon source for bacterial cellulose production has never been reported previously. Moreover, this capacity offers the possibility of restoration of a characteristic industrial waste (a PAH) into a value‐added polymer with multiple application possibilities in biotechnology (Abitbol *et al*., 2016). Cellulose production by strain N1B does not require exhaustive oxygenation of the cultures because the strain is adapted to microaerophilic growth (Martirani‐Von Abercron *et al*., 2017). This is facilitated by the high affinity for oxygen of the oxygenases encoded in the *dbd* cluster, acquired by the strain through lateral gene transfer during enrichment on naphthalene (Hirano *et al*., 2006, 2007). The complex and precise architecture of the cellulose matrix organized around the naphthalene crystal suggests an important role in cellulose degradation, probably related to the optimization of processes such as mass transfer, adsorption of the PAH to the cellulose matrix to diminish the distance of the cell to the substrate and protection against PAH toxicity. The identification of the pathways from naphthalene to cellulose opens up the possibility of constructing genome‐scale metabolic models to optimize substrate utilization and polymer production, which is underway in our laboratory. Furthermore, the manipulation of the hydrocarbon degradation capacity of the strain could be manipulated to increase the range of substrates to be metabolized by the composite PAH degradation pathway identified in the strain, broadening the possibilities of the exploitation of the strain's metabolism to the transformation of contaminants into a value‐added polymer.

## Experimental procedures

### Materials and standard procedures

All the chemicals used in this study were from Fluka (Neu Ulm, Germany), Sigma‐Aldrich Labor Chemie (Steinheim, Germany), Perkin Elmer Life Science (Boston, MA, USA) or Merck Serono (Darmstadt, Germany). Restriction enzymes were from New England Biolabs (Frankfurt am Main, Germany). T4 DNA ligase was purchased from Roche (Grenzach‐Wyhlen, Germany). The plasmids were isolated with ‘Qiapreps spin plasmid kit’ of Qiagen (Hilden, Germany). PCRs were purified with ‘Qiaquick Gel Extraction Kit’ of Qiagen. Oligonucleotides were synthesized by Sigma‐Aldrich. Transformation, PCR amplification with Expan High Fidelity (Roche) and protein analysis were performed according to standard protocols (Ausubel *et al*., 1991).

### Bacterial strains, media and culture conditions

The strain *Starkeya* sp. N1B used in this study was isolated from a hydrocarbon‐polluted aquifer after enrichment with naphthalene crystals as sole carbon source under microaerophilic conditions (Martirani‐Von Abercron *et al*., 2017). The strain was routinely grown under microaerophilic conditions a 28°C in 50 ml static bottles filled with 40 ml of a modified non‐reduced Widdel Mineral Medium (WMM) prepared as described (Pacheco‐Sánchez *et al*., 2017). The final WMM medium contained 30 mM 3‐(N‐morpholino) propanesulfonic acid (MOPS) as buffer, 8 mM potassium nitrate, 17.1 mM NaCl, 6.7 mM KCl, 5.9 mM CaCl_2_, 4.7 mM NH_4_Cl, 2 mM MgCl_2_, 1.44 mM KH_2_PO_4_, 1 mM Na_2_SO_4_ and was supplemented with 12 μl of a vitamin solution (Widdel and Bak, 1992), 40 μl of the SL10 trace element solution (Widdel *et al*., 1983) and 40 μl of a selenium‐tungsten solution (Widdel, 1988) per 40 ml medium. As carbon source, a naphthalene crystal (weighing between 10 and 40 mg) was added to each bottle. The nalidixic acid and kanamycin antibiotics were added to the cultures. The bottles were initially prepared under a nitrogen atmosphere and sealed with Viton stoppers. After inoculation, a 30‐gauge needle was inserted through the Viton stopper to allow a slow rate oxygen flow‐through into the culture vessels. In the absence of an inoculum, the oxygen only reached saturation after 5–7 days (Martirani‐Von Abercron *et al*., 2017). In the presence of growing bacteria, the rate of oxygen consumption established microaerophilic conditions (below 7 ppm of dissolved oxygen). When required, naphthalene was provided dissolved (10 g l^−1^) in an upper layer of 2 ml of 2,2,4,4,6,8,8‐heptamethylnonane (HMN). Aerobic cultivation of strain N1B was carried out in 100 ml Erlenmeyer flasks filled with 10 ml of WMM supplemented with a naphthalene crystal and shaken at 100 rpm. When appropriate, antibiotics were added at the following concentrations (in micrograms per millilitre): kanamycin, 5; nalidixic acid, 10; streptomycin, 50; spectinomycin, 100. Nalidixic acid and kanamycin were routinely used for selection of strain N1B or its derivatives, since this strain is naturally resistant to these antibiotics. The *E. coli* strains were grown on LB supplemented with the appropriate antibiotics at the following concentration (in micrograms per millilitre): rifampin, 30; chloramphenicol, 30; ampicillin, 100; streptomycin, 50 and spectinomycin, 100.

### Random mutagenesis and identification of Mini‐Tn5 insertion

Transposon mutagenesis with mini‐Tn5‐ΩSm (de Lorenzo *et al*., 1990) was performed by triparental mating. The recipient (*Starkeya* sp. strain N1B) was grown aerobically for 48 h in WMM supplemented with 25 mM glucose as carbon source and the antibiotics nalidixic acid and kanamycin; the donor [*E. coli* CC118‐λpir harbouring the mutagenic suicide vector pUT‐ΩSm/Sp which confers resistance to streptomycin (Sm) and spectinomycin (Sp)] (de Lorenzo *et al*., 1990) and helper [*E. coli* HB101 (pRK600)] (Kessler *et al*., 1992) strains were grown in LB with the appropriate antibiotics until they reached an OD_660_ = 0.5. 25 ml of the recipient strain was collected and mixed with 0.5 ml of the donor and helper strains. The cells were collected by centrifugation for 10 min at 8800 *g* at room temperature, re‐suspended in 100 μl of fresh LB and spotted on a 0.22 μm pore cellulose filter on an LB plate. After overnight incubation at 30°C, the cells were scraped off the filter and re‐suspended in 3 ml of WMM, and serial dilutions were plated on selective WMM supplemented with 1/10 LB as the carbon source and the nalidixic acid, kanamycin, plus streptomycin and spectinomycin antibiotics for the selection of transconjugants. To select for mutants impaired in the use of naphthalene as the carbon source, each transconjugant was replicated in WMM plates supplemented with naphthalene from the vapour phase by placing a naphthalene crystal on the upside down lid. To determine the DNA sequence targeted in the selected transposon mutant, we cloned the chromosome fragment carrying the Sm/Sp resistance cassette. To that end, the chromosomal DNA of the mutant was digested with XhoI and cloned into pBlueScript digested with the same enzyme. The Sm/Sp resistant clones were sequenced to identify the transposon insertion site.

### Site‐specific homologous *bcsA* gene inactivation

The Δ*bcsA* deletion mutant was generated by double homologous recombination using a pKNG101 (Kaniga *et al*., 1991) plasmid derivative as described (Pacheco‐Sánchez *et al*., 2017). Briefly, a 2.3 Kb fragment encompassing the 5′ 851 first nucleotides of *bcsA* and the preceding 1449 bp sequence was amplified from strain N1B genome DNA and cloned in pCR2.1‐TOPO^R^ to render plasmid pTOPO:bcsN; a 2.04 Kb fragment encompassing the last 3657 nucleotides of *bcsA* and the following 1382 bp sequence was cloned in pMBL‐T™ to render pMBL:bcsB. This latter plasmid was digested with SmaI (cutting the *bcsA* sequence) and XbaI (cutting the polylinker sequence) to obtain a DNA fragment encompassing the last 138 nucleotides of *bcsA* and the following 1382 bp sequence, and the resulting fragment was cloned downstream the *bcsA* fragment in pTOPO:bcsN. This generated a 1.2 kb deletion of *bcsA* in pTOPO:bcsNΔAbcsB. The fragment covering the deleted *bcsA* gene and its flanking sequences was then subcloned in pKNG101 as ApaI‐SpeI fragment to obtain pKNGΔbcsA. This plasmid was used to deliver the mutations to strain N1B chromosome by RP4‐mediated mobilization, as follows: the recipient *Starkeya* sp. strain N1B, the *E. coli* donor strain CC118λpir bearing pKNGΔbcsA and *E. coli* HB101 (pRK600) helper strain were grown, mixed and incubated on a filter for conjugal DNA transfer as described above. After 24 hours, the filter was transferred into 3 ml of WMM and the cells were washed off by vigorous vortexing and serial dilutions were plated on WMM 1/10 LB supplemented with nalidixic acid, kanamycin plus streptomycin to select transconjugants bearing a cointegrate of pKNGΔbcsA in the chromosome. Streptomycin‐resistant transconjugants were analysed by PCR with primers flanking *bcsA* deletion. Those in which both the wild type and the mutated gene products were amplified were selected and cultured in WMM 1/10 LB with nalidixic acid and kanamycin for 12–16 h to promote the second crossover and the allelic exchange to occur. To select double recombinants, colonies were plated on WMM 1/10 LB with 10% (w/v) sucrose. Streptomycin‐sensitive/sucrose‐resistant colonies were analysed by PCR to confirm the gene deletion. The correct exchange of the deleted region was confirmed by PCR analysis and Southern blot.

### DNA sequencing

Sanger DNA sequencing was performed by the DNA Sequencing Service at the López‐Neyra Parasitology and Biomedicine Institute (IPBLN), (Granada, Spain). Whole genome sequencing with Illumina MySeq platform and basic bioinformatics analysis was carried out at Era7 Information Technologies S.L (Granada, Spain).

### Scanning electron microscopy (SEM)

Specimen preparation and SEM were performed at the Scientific Instrumentation Centre (CIC) of the University of Granada. Biofilm samples from microaerophilic cultures growing on naphthalene were gently collected from the bottles after draining the medium. Then, the samples were fixed for 2 h at 4°C in 2.5% glutaraldehyde prepared in cacodylate buffer, pH 7.4. The fixed samples were rinsed three times (15 min each) with the same buffer at 4°C and incubated for 1 h with 1% osmium tetroxide at room temperature, rinsed three times (5 min) with water and dehydrated in an increasing ethanol concentration gradient from 50% to 100%. Samples were further desiccated with carbon dioxide in a Leica EM CPD300 critical point dryer according to Anderson (1951). The samples were carbon coated in an EMTECH K975X evaporator and examined in a Zeiss SUPRA40VP scanning electron microscope equipped with a Schottky type emission gun.

### Cellulose characterization

Atomic force microscopy (AFM) was used to determine the morphology of the surface of BC freeze‐dried membranes. AFM images were obtained in tapping mode using a Nanoscope IIIa scanning probe microscope (MultimodeTM, Digital instruments, Santa Barbara, CA, USA) with an integrated force generated by cantilever/silicon probes, applying a resonance frequency of about 180 kHz. Cantilevers had a tip radius of 5–10 nm and were 125 μm long. The morphology of the BC membranes was studied in tapping mode. Samples of the membranes were cut into 5 × 5 mm^2^ and stuck in mica. Attenuated total reflectance–Fourier transform infrared (ATR‐FTIR) spectroscopy was used to identify the characteristic functional groups of the samples. Spectra were recorded using a Nicolet Nexus spectrophotometer provided with MKII Golden gate accessory (Specac) with diamond crystal at a nominal incidence angle of 45° and ZnSe lens. Measurements were recorded after averaging 32 scans with a resolution of 4 cm^−1^ in the range 4000 to 650 cm^−1^. Lyophilized samples were dried for 24 h in an oven at 60 °C before X‐ray diffraction (XRD) and ATR‐FTIR analysis. XRD patterns of different samples were measured using PHILIPS X'Pert Pro diffractometer, in theta–theta configuration secondary monochromator with CuKα (λ = 0.154 nm) and a solid state pixel detector, operating at 40 kV with a filament of 40 mA. The X‐ray diffractrograms were collected in the region 2θ = 5° to 50°, where θ is the angle of incidence of the X‐ray been on the sample. The crystallinity index (CI^XRD^) was calculated using the empirical method described by (Segal *et al*., 1959) using the Eq. (1).

(1) $${\text{CI}}^{\mathrm{XRD}}=\frac{I_{200}-I_{\mathrm{am}}}{I_{200}}$$

where *I*_200_ is the maximum intensity of the (200) lattice diffraction at ~ 2θ = 22.7° and *I*_am_ is the intensity scattered by the amorphous part of the sample (the location of the amorphous material intensity considered in this work was at ~ 2θ = 18°).

### Sequence analysis and deposition

We used SPoctopus (Viklund *et al*., 2008) and DAS (Cserzo *et al*., 1997) for transmembrane segment prediction, SignalP 4.1 (Nielsen, 2017) for signal peptide prediction, Blastp (Johnson *et al*., 2008) for sequence comparison with the databases and the tool set available at the JGI‐IMG (Chen *et al*., 2017) for gene neighbourhood analysis.

The sequence of some *dbd* gene products was already deposited in GenBank with ID MF537652 to MF537654. The whole *dbd* cluster sequence and the remaining clusters involved in naphthalene degradation have been deposited in GenBank with ID MK330903 to MK330905.

The sequences for the cellulose synthesis operon have deposited in GenBank with ID MK350252.

## Conflict of interest

The authors declare that the research was conducted in the absence of any commercial or financial relationships that could be construed as a potential conflict of interest.

## Supporting information


**Fig. S1. (**A) Biofilm developed by *Starkeya* sp. strain N1B when growing with naphthalene dissolved in HMN as sole carbon source.
**Fig. S2.** Aerobic naphthalene degradation pathway identified in strain N1B. The substrate and pathway intermediates are indicated.
**Fig. S3.** Scheme of the gene organization and genetic context of the *bcs* cluster in strain N1B, and homology with the equivalent region in the close relative strain *Ancylobacter rudongense* that lacks the *bcs* cluster.
