# Supplementary material for: Bacterial nanocellulose production from naphthalene
Source: Microb Biotechnol. 2019 May 14;12(4):662–76. doi: 10.1111/1751-7915.13399 (PMC6559018; doi:10.1111/1751-7915.13399)
Supplement: Supplementary file 1 — Fig. S1. (A) Biofilm developed by Starkeya sp. strain N1B when growing with naphthalene dissolved in HMN as sole carbon source. Fig. S2. Aerobic naphthalene degradation pathway identified in strain N1B. The substrate and pathway intermediates are indicated. Fig. S3. Scheme of the gene organization and genetic context of the bcs cluster in strain N1B, and homology with the equivalent region in the close relative strain Ancylobacter rudongense that lacks the bcs cluster. [file MBT2-12-662-s001.pdf]

Supporting information:

Bacterial nanocellulose production from naphthalene.

Patricia Marín[1], Sophie Marie Martirani-Von Abercron[1], Leyre Urbina[2], Daniel Pacheco-Sánchez[1], Mayra Alejandra Castañeda-Cataña[1], Aloña Retegi[2], Arantxa Eceiza[2] & Silvia Marqués[1]

[1]Estación Experimental del Zaidín, Department of Environmental Protection, Consejo Superior de Investigaciones Científicas, Granada, Spain.
[22]Materials + Technologies Research Group (GMT), Department of Chemical and Environmental Engineering, Faculty of Engineering of Gipuzkoa, University of the Basque Country, Pza Europa 1, Donostia-San Sebastian, 20018, Spain

*Author for correspondence: Silvia Marqués
Estación Experimental del Zaidín, CSIC
C/. Profesor Albareda nº1
E-18008 Granada, Spain
silvia@eez.csic.es
Tel: +34 958 181600
Fax: +34 958 129600

This file contains 3 supporting figures.

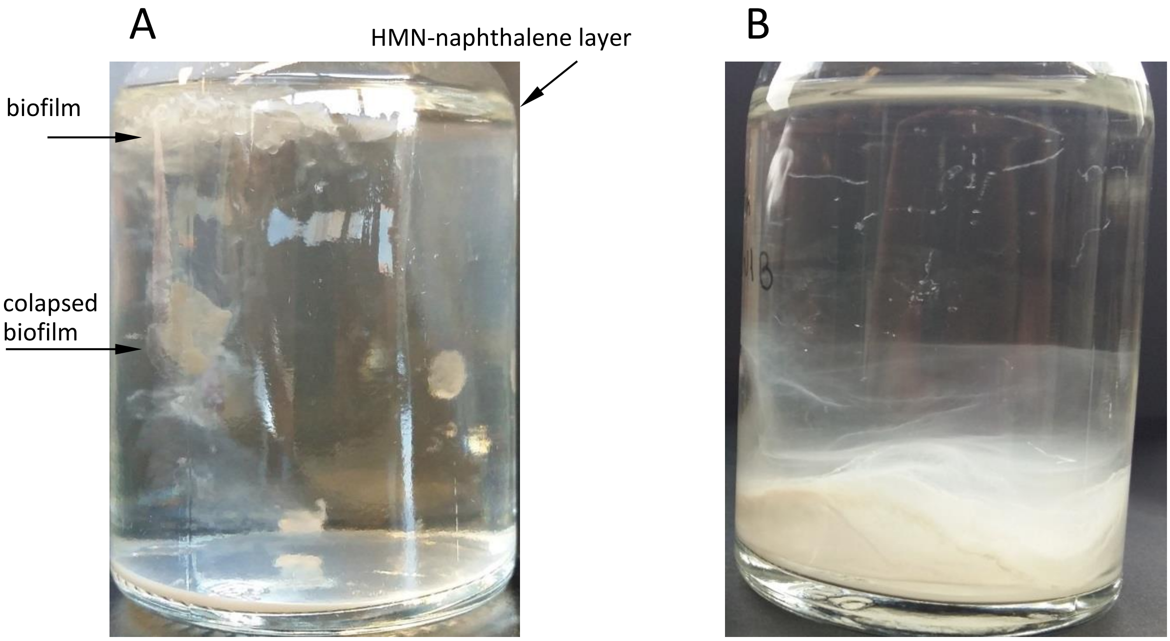

Supporting Figure S1

A) Biofilm developed by strain *Starkeya* sp. strain N1B when growing with glucose as sole carbon source. B) Biofilm developed by strain *Starkeya* sp. strain N1B when growing with naphthalene dissolved in HMN as sole carbon source.

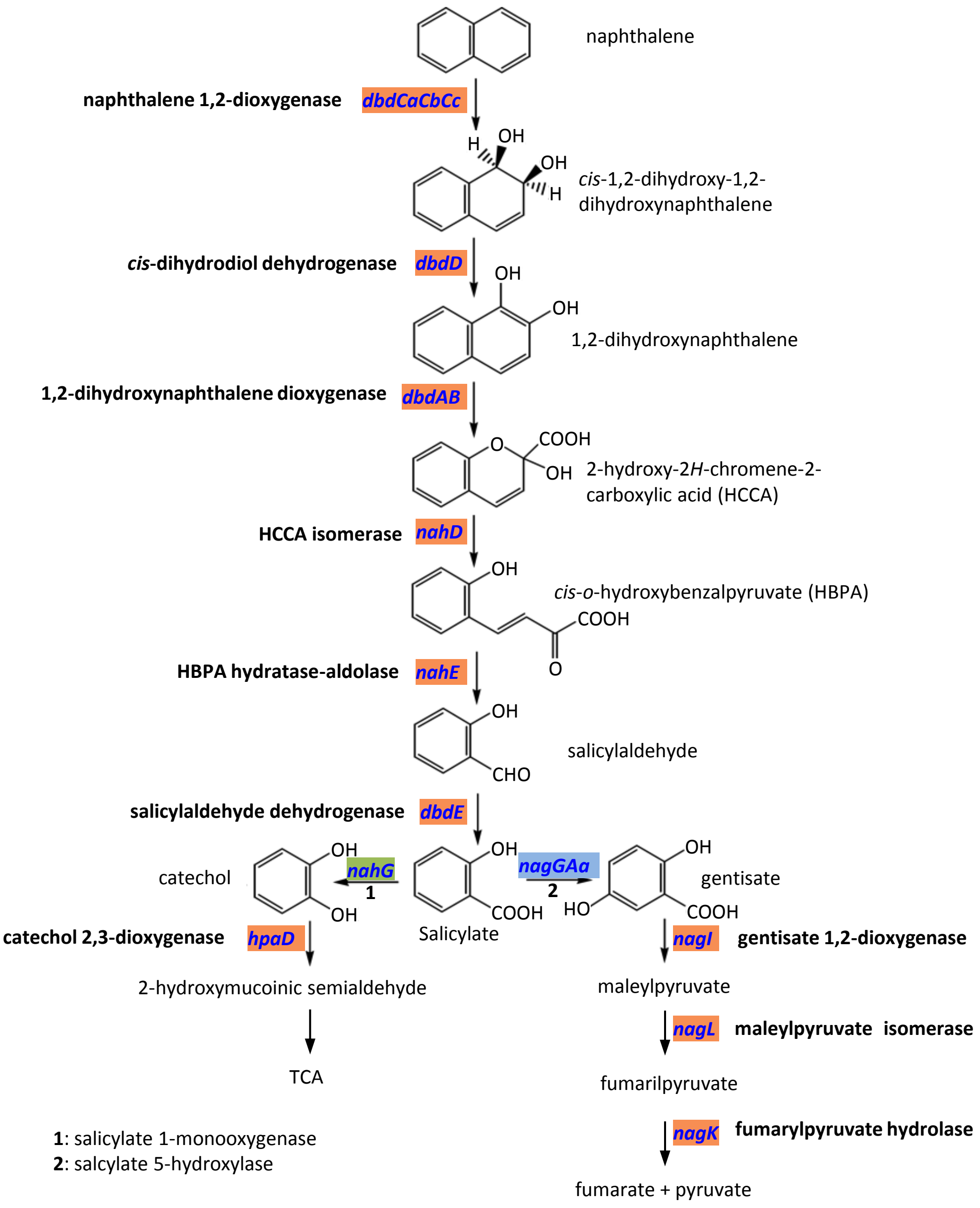

Supporting Figure S2

Proposed aerobic naphthalene degradation pathway identified in strain N1B. The substrate and pathway intermediates are indicated. The enzyme names are in bold. The gene names are coloured according to the code in Fig. 5.

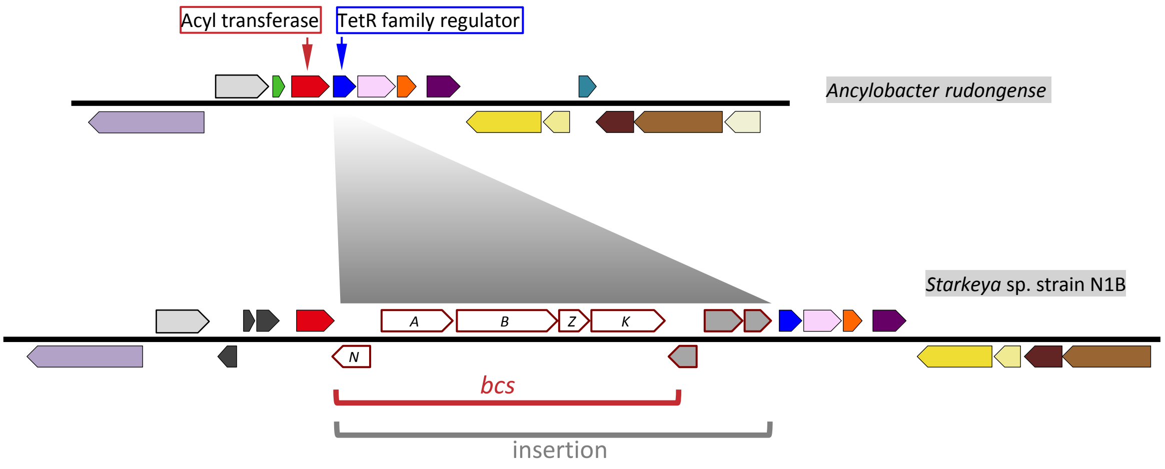

Supporting Figure S3. Scheme of the gene organization and genetic context of the *bcs* cluster in strain N1B, and homology with the equivalent region in the close relative strain *Ancylobacter rudongense* that lacks the *bcs* cluster. Genes with the same colour are homologuous in the two strains. The genes flanking the proposed insertion are shown in red (coding for an acyl transferase) and blue (coding for a TetR family regulator). The *bcs* genes are shown as red-framed white arrows. The remaining genes in the proposed insertion (coding for LTTR, MFS, aldo/keto reductase) are shown as red-framed grey arrows.
